# A flexible proximity sensor formed by duplex screen/screen-offset printing and its application to non-contact detection of human breathing

**DOI:** 10.1038/srep19947

**Published:** 2016-01-22

**Authors:** Ken-ichi Nomura, Ryosaku Kaji, Shiro Iwata, Shinobu Otao, Naoto Imawaka, Katsumi Yoshino, Ryosuke Mitsui, Junya Sato, Seiya Takahashi, Shin-ichiro Nakajima, Hirobumi Ushijima

**Affiliations:** 1 Flexible Electronics Research Centre, National Institute of Advanced Industrial Science and Technology (AIST), 1-1-1 Higashi, Tsukuba, Ibaraki 305-8565, Japan; 2 Intelligent Systems Research Institute, National Institute of Advanced Industrial Science and Technology (AIST), 1-1-1 Umezono, Tsukuba, Ibaraki 305-8568, Japan; 3 Shimane Institute for Industrial Technology (SIIT), 1 Hokuryo-cho, Matsue, Shimane 690-0816, Japan; 4 Japan Aviation Electronics Industry, Ltd. (JAE), 3-1-1 Musashino, Akishima, Tokyo 196-8555, Japan

## Abstract

We describe a flexible capacitance-type sensor that can detect an approaching human without contact, fabricated by developing and applying duplex conductive-ink printing to a film substrate. The results of our calculations show that the difference in size between the top and bottom electrodes of the sensor allows for the spatial extension of the electric field distribution over the electrodes. Hence, such a component functions as a proximity sensor. This thin and light device with a large form factor can be arranged at various places, including curved surfaces and the back of objects such that it is unnoticeable. In our experiment, we attached it to the back of a bed, and found that our device successfully detected the breathing of a subject on the bed without contacting his body. This should contribute to reducing the physical and psychological discomfort among patients during medical checks, or when their condition is being monitored.

Technology to sense human vital signs is important to maintaining and promoting health. Such sensing data helps prevent diseases as well as accidents, thus contributing to a reduction in medical costs. For example, sleep apnoea syndrome threatens our health. To address this condition, a medical check is often performed while patients are asleep in bed^1^. Various sensors are attached to the patients’ bodies for this purpose, causing electric cables and wires to be scattered around them, and rendering them uncomfortable. Another candidate method to obtain vital signals involves the use of wearable smart devices, such as a smart watch^2^,^3^,^4^. Smart watches do not need cables or wires. If worn by patients while sleeping, these devices can easily obtain the relevant vital data. At this stage of technological development in the area, the use of smart clothes^5^,^6^ is also a potential method to obtain vital signals. Such devices should become effective tools to easily obtain our vital signals in the near future.

However, as a matter of course, smart devices are designed on the assumption that they are worn. We tend to feel a bit strange if such devices are in contact with our bodies while sleeping. Moreover, there is a possibility that we may forget to put the device on before going to bed. Furthermore, wearable devices are unsuitable for many people. For example, it is difficult for an elderly person suffering from dementia or other mental disorders to handle such devices. They can be assisted in putting such devices on, but they may remove them while sleeping due to discomfort. Analogous reasoning applies in the case of toddlers. Thus, the use of non-contact-type sensors that can be arranged such that they are imperceptible to the patient is recommended.

One candidate to measure vital signs that solves the above-mentioned issues is a sensor based on electromagnetic waves. A microwave Doppler sensor is a typical device of this sort^7^. This type of the sensor is often attached to a wall or ceiling, and radiates microwaves to human bodies. The duration of breathing can be calculated by measuring and analysing the reflected waves^8^. However, there are certain problems in such sensor systems. First, the sensor is easily noticeable. A person in a delirious state^9^ may try to break it because he/she might feel uncomfortable at being surveilled. Second, the sensor system is fabricated on rigid substrates. This limits its potential installation locations. For example, it cannot be attached to a curved surface. Furthermore, such a substrate has low impact resistance and can break easily. Third, such a sensor component is expensive because it is fabricated by conventional vacuum and photolithographic processes, and the vacuum processes require considerable amounts of energy to attain and maintain low pressure. This increases the cost of electricity. Moreover, photolithography is a “subtractive” process, where some material is coated on the surface of a substrate, and unnecessary parts are subsequently removed by etching. This causes a rise in the cost of materials. Fabrication machines are also quite expensive, and therefore increase facility investment cost.

Film-type flexible devices have attracted considerable attention because they can overcome the problems mentioned above; for, example, skin prosthesis devices and multifunctional wearable devices for diagnosis and/or therapy of movement disorders have been reported so far^10^,^11^,^12^,^13^,^14^,^15^,^16^. Thin films can provide impact-resistant and high form factor devices. Further, thin components can be attached to a wall, ceiling, floor, carpet, bed, etc., such that they are unnoticeable. It would be even more fascinating if such devices were fabricated using printing techniques. Printing is not necessary in the application of a vacuum process to pattern formation. It is an “additive” method that forms patterns only where we want. These facts allow us to save on fabrication cost. Of the various printing methods, screen printing is the most widely used. Electrodes for devices, such as solar cells^17^,^18^,^19^,^20^,^21^,^22^,^23^ and capacitors^24^,^25^,^26^,^27^,^28^,^29^, are often formed by this method.

To address the issues summarised in the foregoing, we describe in this paper our development of a film-based printed capacitance-type proximity sensor. Although a similar flexible proximity sensor fabricated using inkjet printing has been reported^30^, our fabrication method is superior especially in short processing time, compared to inkjet printing. Namely, we arranged the electrodes of the sensor at the top and bottom surfaces of a polyethylene terephthalate (PET) film fabricated by a novel yet simple duplex printing technique, which is a combination of screen printing and screen-offset printing^31^,^32^. Although this sensor can be simply used to detect an approaching person, it can also be applied to detect breathing. In this paper, we detail our printing method, and describe the properties and impact of our proximity sensor by analysing its sensitivity. Furthermore, we show that our sensor can accurately detect the duration of breathing of a person, as an instance of its many promising applications.

## Results

### Fabrication of sensor by combination of screen/screen-offset printing

**Figure 1:**
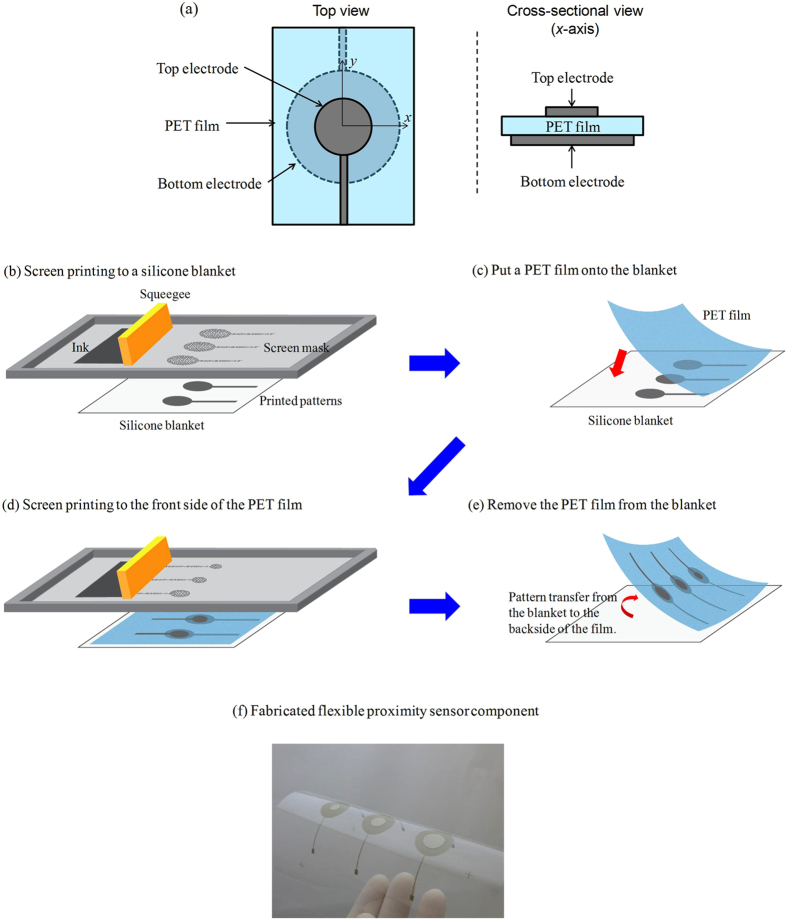
Fabrication of the film-type proximity sensor. (**a**) Schematic diagramme of the structure of our novel film-type proximity sensor. (**b–e**) Fabrication procedure of the proximity sensor: Screen printing a circular-shaped bottom Ag electrode on a silicone blanket (**b**), pressing a PET film onto the blanket (**c**), printing a circular top electrode at the top of the film using another screen mask (**d**), and peeling the film off the blanket, followed by thermal treatment (**e**). (**f**) An example of the sensor patterns formed by the above-mentioned procedure.

Figure 1a shows a schematic diagramme explaining the film-type proximity sensor that we developed. This is a capacitance-type sensor with two electrodes of different sizes at the top and the bottom of the PET substrate, as detailed in the following section. The two-sided electrodes of the proximity sensor were fabricated by a combination of screen printing and screen-offset printing^31^,^32^. Here, we explain screen-offset printing. In this sort of printing, we first performed screen printing on a blanket made of silicone. The ink on the blanket was subsequently transferred to a substrate. The silicone blanket absorbs solvents in ink^33^. Thus, on the blanket, the interface between the air and the ink became dry, whereas that between the ink and the blanket was wet, and is called “dry ink”^34^. Because of this, the pattern formed on the blanket could be transferred to a final substrate. Furthermore, the ink-solvent absorption of the silicone blanket prevents the patterns from spreading or increasing their width. Thus, although the patterns of our current proximity sensor are comparatively large, we can obtain finer patterns of widths of a few tens of micrometres through screen-offset printing^32^, which are very difficult to form using conventional screen printing.

Figure 1b–f show the fabrication of the proximity sensor. The circular-shaped bottom silver (Ag) electrode pattern was first screen printed on a silicone blanket (Fig. 1b). The pattern on the blanket was then covered with a PET film by pressing with a hand rubber roller (Fig. 1c). Subsequently, the screen mask was changed to print the top electrode, and another circular Ag pattern was screen printed at the top of the PET film (Fig. 1d). The film was then peeled off the blanket. In this case, the bottom electrode pattern was transcriptionally formed at the bottom of the film from the blanket (Fig. 1e). Following Ag pattern formation, the ink on the film was thermally treated at 130 °C for five min in air. Figure 1f shows an example of the pattern formed by the above-mentioned procedure. If conventional printing is employed, the substrate needs to be taken out of the stage following the printing of the bottom electrode, and subsequent thermal treatment of the electrode needs to be undertaken. The top electrode is then printed and annealed. On the contrary, our novel method does not require the removal of the bottom electrode-printed film from the printing stage. This allows for a shorter processing time as well as easy alignment of the top and bottom electrodes.

### Sensing mechanism of the proximity sensor

As mentioned above, our proposed sensor is capacitance type. In our experiment, we employed the chemical impedance analyser (Hioki, IM3590, Nagano, Japan) using the auto-balancing bridge method for capacitance measurement. Alternating current (AC) voltage (5 V, 200 kHz) was applied to the top electrode, while the voltage of the bottom electrode was 0 V. Capacitance was measured by monitoring the current flowing to the bottom. When voltage was applied, electric lines of force, or the electric field, appeared between the top and bottom electrodes. In case the two electrodes are different in size, the electric lines of force can intentionally be leaked from the sensor, as detailed in the “Discussion” section. The human body contains a large amount of water as a conductive material in a frequency range of the order of kilohertz^28^. If a conductive human body approaches a sensor, the body obstructs part of the electric lines of force between the bottom and the top electrode. This results in reduced current flow to the bottom electrode, and hence reduces the apparent capacitance of the sensor. Thus, our sensor can respond to a human body and detect an approaching human. We can see the reduction in capacitance in our experimental demonstration (see Supplementary Movie).

### Dependence of sensing property on sensor electrode size

**Figure 2:**
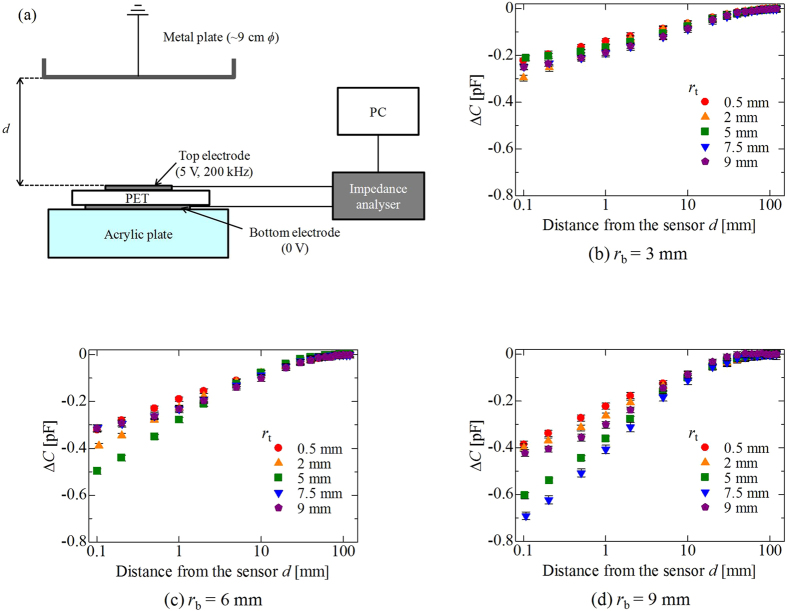
Analyses of sensor properties. (**a**) Schematic diagramme of a system used to test our proximity sensor. (**b**–**d**) Dependences of Δ*C* on distance *d* and top electrode radius *r*_t_, obtained for the sensor with bottom electrode radii *r*_b_ of 3 (**b**), 6 (**c**), and 9 mm (**d**), respectively.

We analysed the performance of our proximity sensor. Figure 2a shows a schematic diagramme explaining the system used for our evaluation experiment. In this system, similarly to the condition in the Supplementary Movie, AC voltage (5 V) with a frequency of 200 kHz was applied to the top electrode, where the voltage of the bottom electrode was 0 V, through electric wires connected to the chemical impedance analyser. The wires were shielded by grounded metal to avoid noise contamination. To facilitate the evaluation, a grounded metal plate with a diameter of ~9 cm was prepared. The capacitance was measured with changing distance *d* between the sensor and the plate. Note that the measurement was performed for ~3 s with a sampling time of ~10 ms at each instance of *d*. In other words, we obtained 300 measurement data items at each *d* (refer to the representative raw data in Supplementary Datasets). We defined the point where the top electrode of the sensor and the plate contacted as *d* = 0. The maximum distance that we could obtain in this system was approximately *d* = 120 mm. In this study, we discuss the change in capacitance Δ*C* as a function of *d*. We measured average capacitance at *d* = 120 mm without arranging the grounded metal plate (*C*_120,w/o_), and will discuss Δ*C*, which was obtained by subtracting *C*_120,w/o_ from average capacitance measured at various *d* values (*C*_*d*_). Furthermore, we obtained the maximum and minimum values of capacitance at *d* = 120 mm without the plate from the 300 data items, referred to as *C*_120,w/o,max_ and *C*_120,w/o,min_, respectively. Similarly, we obtained maximum and minimum capacitance values at each *d*, referred to as *C*_*d*,max_ and *C*_*d*,min_, respectively. In the later described figures discussing Δ*C*, the upper level of error bars indicates *C*_*d*,max_−*C*_120,w/o,min_, whereas the lower level represents *C*_*d*,min_−*C*_120,w/o,max_. Further, note that at distances greater than ~100 mm, Δ*C* was near zero in these experimental conditions, indicating that the sensor was insensitive at this distance.

**Figure 3:**
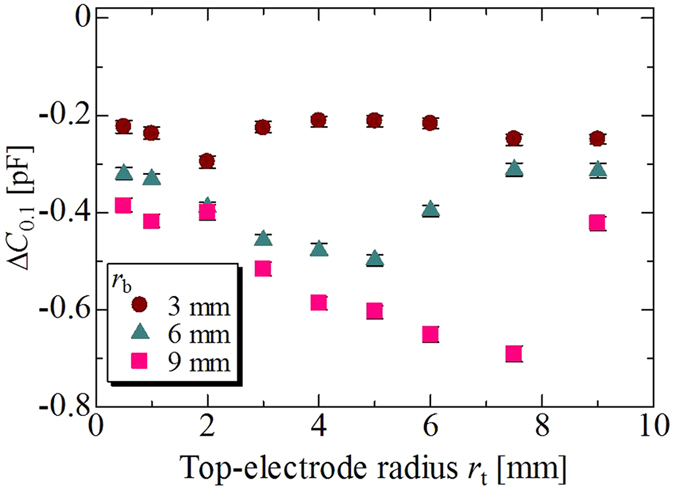
Capacitance change Δ*C*_0.1_ when the metal plate is in the vicinity (*d* = 0.1 mm) of the sensor.

We first performed measurements with sensors placed flat using various top *r*_t_ and bottom electrode radii *r*_b_. In this study, *r*_t_* = *0.5–9 (0.5, 1, 2, 3, 4, 5, 6, 7.5, and 9 mm) and *r*_b_ = 3, 6, and 9 mm. Figure 2b–d shows Δ*C* as a function of *d*, obtained for values of *r*_b_ of 3, 6, and 9 mm, respectively. We plotted the representative data of *r*_t_ = 0.5, 2, 5, 7.5, and 9 mm to keep the figures clean. We found that the value of Δ*C* decreased with a reduction in *d* for all conditions, but its degree of change was significantly dependent on *r*_t_ and *r*_b_. To explicate these dependences, Fig. 3 shows the value of Δ*C*_0.1_ ( = *C*_0.1_ – *C*_120,w/o_) as a function of *r*_t_, where *C*_0.1_ was the average capacitance at *d* = 0.1 mm. For all *r*_b_, the value of Δ*C*_0.1_ first decreased with increasing *r*_t_, reached the minimum value Δ*C*_0.1,min_, and increased when *r*_t_ ≥ *r*_b_, indicating that the sensor response deteriorated when the structure of the sensor changed to a parallel-plate capacitor. This implies that the most appropriate *r*_t_ existed corresponding to *r*_b_. Moreover, we found that Δ*C*_0.1,min_ decreased with increasing *r*_b_. These results indicate that a larger value of *r*_b_ yielded a more sensitive response from the sensor. Under the conditions of this experiment, the combination of *r*_t_ = 7.5 mm and *r*_b_ = 9 mm yielded the greatest change in Δ*C*_0.1_.

**Figure 4:**
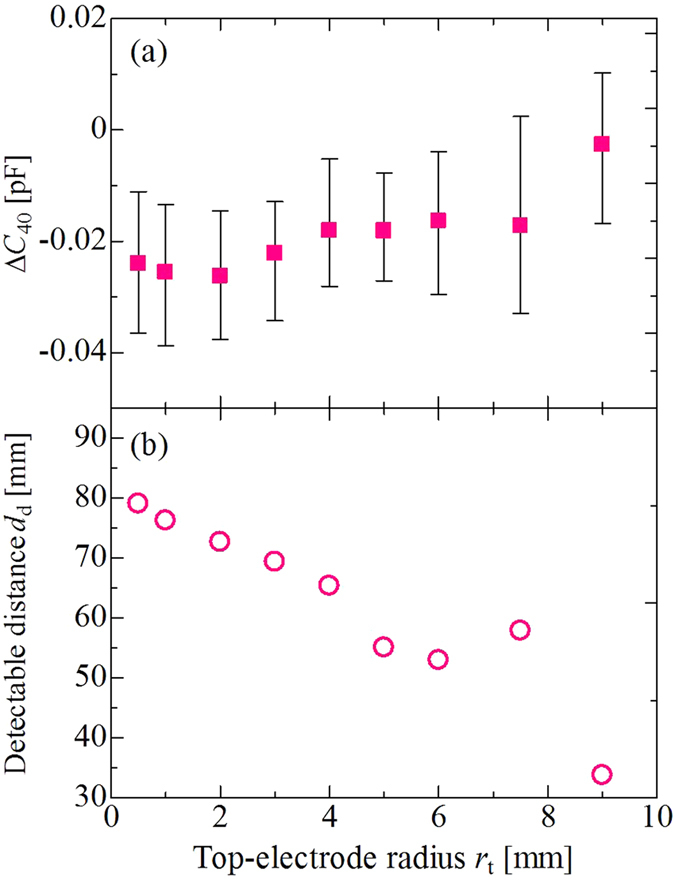
Capacitance change and detectable distance. (**a**) Capacitance change Δ*C*_40_ for the sensor with *r*_b_ = 9 mm. The metal plate was at *d* = 40 mm. (**b**) Detectable distance (limit of detection) *d*_d_.

On the contrary, interesting results were observed regarding sensitivity at *d* of the order of centimetres. As a typical example, Fig. 4(a) shows the dependence of Δ*C*_40_ ( = *C*_40_ – *C*_120,w/o_) on *r*_t_ in the case of *r*_b_ = 9 mm, where *C*_40_ is capacitance at a distance of 40 mm. We found that Δ*C*_40_ assumed its minimum value when *r*_t_ = 2 mm, and deteriorated with increasing values of *r*_t_. This tendency was different from that of Δ*C*_0.1_, shown in Fig. 3. For further analysis, we attempted to estimate the detectable distance *d*_d_ by employing the definition proposed by the International Union of Pure and Applied Chemistry (IUPAC)^36^,^37^. According to the definition, we needed to estimate fluctuation level –*kσ* of our sensor system, where *σ* is the standard deviation of the signal, and character *k* is a numerical factor that determines the confidence level. In general, the preferred value is *k* = 3 because it guarantees a high confidence level of 99.6% theoretically, and 90% in practical scenarios^37^. From this definition, we could determine the distance where Δ*C* was lower than –3*σ*, as *d*_d_. The noise deviation level 3*σ* of our system was ~8 fF; we employed 8 fF as the 3*σ* value. The estimated value of *d*_d_ as a function of *r*_t_ is shown in Fig. 4(b). *d*_d_ decreased with increasing *r*_t_, with a maximum value of 79 mm, in cases where *r*_t_ = 0.5 mm.

**Figure 5:**
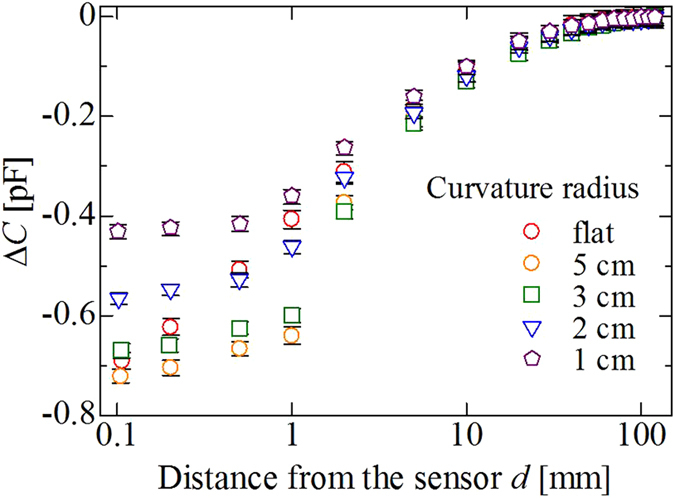
Dependence of Δ*C* on a curvature radius obtained for the sensor with *r*_t_ = 7.5 mm and *r*_b_ = 9 mm.

Another interesting phenomenon was as follows: We compared the values of Δ*C* as a function of *d* in the case where the sensor was curved, with curvature radii *r* of 1–5 cm. The sensor sheet was curved around an axis parallel to the *x*-axis, as shown in Fig. 1, by attaching it to a curvy acrylic plate. Note also that we defined the point where the sensor electrode and the metallic plate touched as *d* = 0. Figure 5 shows the results obtained using the sensor with *r*_t_ = 7.5 mm and *r*_b_ = 9 mm. When *d* ranged on the order of centimetres, the difference in the value of Δ*C* between the curved sensors and the flat one seems small. This indicates the high likelihood that the sensor sheet can be placed on various curved surfaces, even if the curvature radius is as small as 1 cm. At the same time, the results were very complicated when *d *~ 1 mm: the change in Δ*C* was large, of the order of *r* = 5, 3, 2, ∞ (flat), and 1 cm. This indicates that we could further enhance the sensor’s ability by curving it on such a distance range. We also found that the value of Δ*C* saturated in the vicinity of the sensor (*d* < 1 mm) for each curved sensor.

### Application of the sensor to a breathing detection system

**Figure 6:**
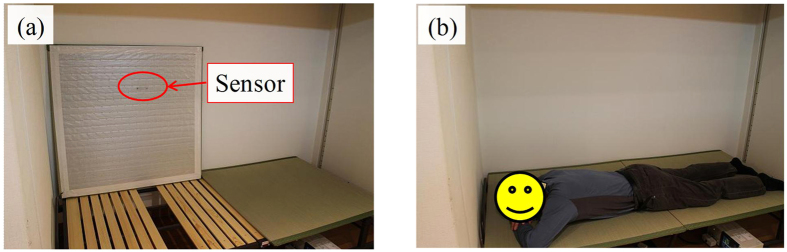
Unnoticed breathing detection system using a *tatami* bed. (**a**) *Tatami* bed and film-type proximity sensor attached to its back. (**b**) Measurement of human breathing performed when subject is lying on the bed.

As a promising application of our novel proximity sensor, we attempted to detect human breathing through it. In this experiment, a Japanese *tatami* bed with a thickness of ~1.5 cm was prepared to perform the demonstration. A proximity sensor with *r*_t_ = 0.5 mm and *r*_b_ = 9 mm was attached to the back of the *tatami* (Fig. 6a). The *tatami* was not made of a conductive material, and the electric field from the sensor could penetrate it. An examinee—one of the authors of this paper: male, 176 cm tall, and weighing 65 kg—lay on the *tatami*, and his chest was brought in the vicinity of the sensor (Fig. 6b). The sensor was connected to an impedance analyser, and capacitance was measured as a function of time.

**Figure 7:**
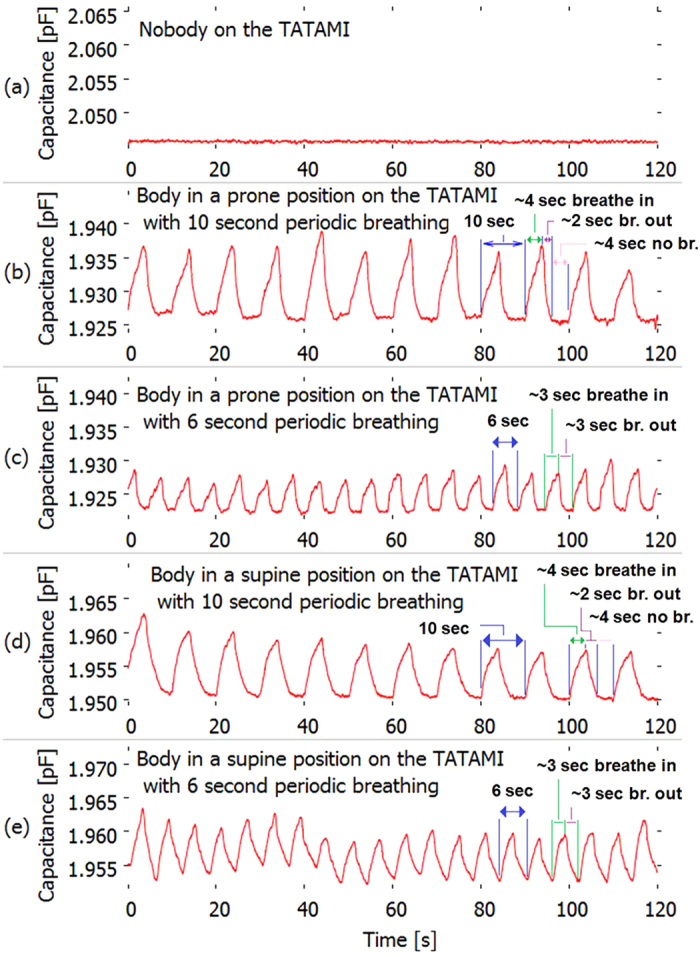
Experimental results for breathing detection. (**a**) Capacitance as a function of time when no one was on the *tatami*. (**b–d**) Capacitance change in the case that the examinee lay on the *tatami*: a prone position with 10-s periodic breathing (~4 s for suction, ~2 s for delivery, and ~4 s for apnoea) (**b**), a prone position with 6-s periodic breathing (~3 s for suction and ~3 s for delivery) (**c**), a supine position with 10-s periodic breathing (~4 s for suction, ~2 s for delivery, and ~4 s for apnoea) (**d**), and a supine position with 6-s periodic breathing (~3 s for suction, ~3 s for delivery) (**e**).

Figure 7 shows the capacitance measured as a function of time in cases where there was no one on the *tatami* (a), where the examinee lay on the *tatami* in a prone position with 10-s periodic breathing (~4 s for suction, ~2 s for delivery, and ~4 s for apnoea) (b), in a prone position with 6-s periodic breathing (~3 s for suction and ~3 s for delivery) (c), in a supine position with 10-s periodic breathing (~4 s for suction, ~2 s for delivery, and ~4 s for apnoea) (d), in a supine position with 6-s periodic breathing (~3 s for suction, ~3 s for delivery) (e). As can be seen from the figures, the number of nominal peaks for the 120 s of measurements was 12 for 10-s breathing (Fig. 7b,d), whereas it was 20 for 6-s breathing (Fig. 7c,e). These numbers are consistent with those obtained for breathing times performed in this experiment. We also found that capacitance increased, dropped, and remained constant during suction, delivery, and apnoea, respectively (Fig. 7b–e). On the other hand, no significant peaks were observed when no one was on the *tatami*. This indicates that capacitance peaks appearing in Fig. 7b–e were due to human breathing.

## Discussion

**Figure 8:**
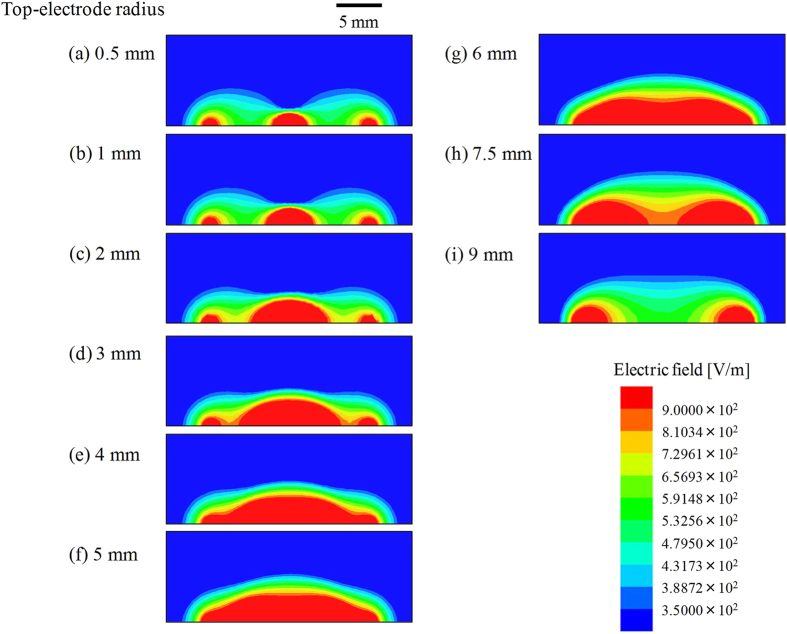
Simulation results of electric field distribution around the sensor where *r*_b_ = 9 mm. *r*_t_ = 0.5 (**a**), 1 (**b**), 2 (**c**), 3 (**d**), 4 (**e**), 5 (**f**), 6 (**g**), 7.5 (**h**), and 9 mm (**i**).

To analyse the dependence of the response of the sensor on electrode size, we calculated the electric field distribution surrounding the flatly arranged sensor using a simulator (Ansys, Ansys HFSS, Pennsylvania, USA). We assumed that AC voltage (5 V, 200 kHz) was applied to the top electrodes. Furthermore, the dielectric constant of the PET film was 3.06, which was experimentally obtained using a frequency response analyser (Solartron Analytical, SI 1260 with 1296 Dielectric Interface, Hampshire, UK). Figure 8 shows the electric field distribution in the vicinity of the sensor with *r*_b_ = 9 mm and *r*_t_ = 0.5 (a), 1 (b), 2 (c), 3 (d), 4 (e), 5 (f), 6 (g), 7.5 (h), and 9 mm (i). Note that the images represent a cross-sectional view, and an *x-z* plane in Fig. 1a is shown. We can see that the electric field was spatially leaked outside the sensor component. In case of a small top electrode, typically *r*_t_ = 0.5 mm, the electric field was concentrated at the centre and the edges of the component. In cases where *r*_t_ = 0.5–6 mm, the region, in red where the electric field *E* was stronger than 9 × 102 V/m, seemed to expand with increasing *r*_t_, especially from the centre of the sensor. This was because the electric field strongly appeared close to the voltage-applied top electrodes. In this condition, the area in red seemed largest when *r*_t_ = 6 mm. However, conversely, its strength seemed to suddenly diminish in case of *r*_t_ ≥ 7.5 mm. We assumed that the electric line of force was difficult to spatially leak outside when an excessively large top electrode was employed. In particular, parallel-plate capacitors (*r*_t_ = 9 mm) strongly confine the electric field to the region between the top and bottom electrodes. If a conductive material was placed where the electric field was strong, a greater response should be expected. The experimental results depicted in plots ■ of Fig. 3 show that the sensor response became satisfactory with increasing *r*_t_ (*r*_t_ ≤ 7.5 mm), and suddenly deteriorated at *r*_t_ = 9 mm. It seems that this tendency was consistent with the simulation results shown in Fig. 8a–i.

**Figure 9:**
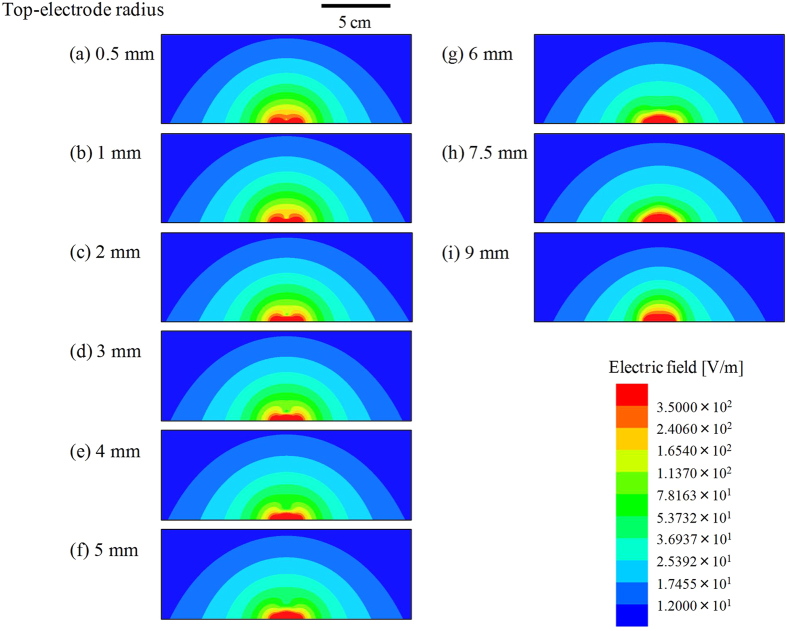
Simulation results of electric field distribution in a long range. (**a–i**) are the same results as shown in Fig. 8a–i, respectively, but the region depicted is larger than that in Fig. 8a–i.

Figure 9a–i show the same results as in Fig. 8a–i, respectively, but the regions depicted are larger than those in Fig. 8a–i. The range of the colour bar also changed. It seems that the distribution of the electric field in a range far from the sensor, for example, the contour line indicating 12 V/m, gradually shrank with increasing *r*_t_. On the contrary, the experimental results shown in Fig. 4 imply that a smaller top electrode yielded better response at regions far from the sensor. This tendency seems to agree with calculation results shown in Fig. 9.

**Figure 10:**
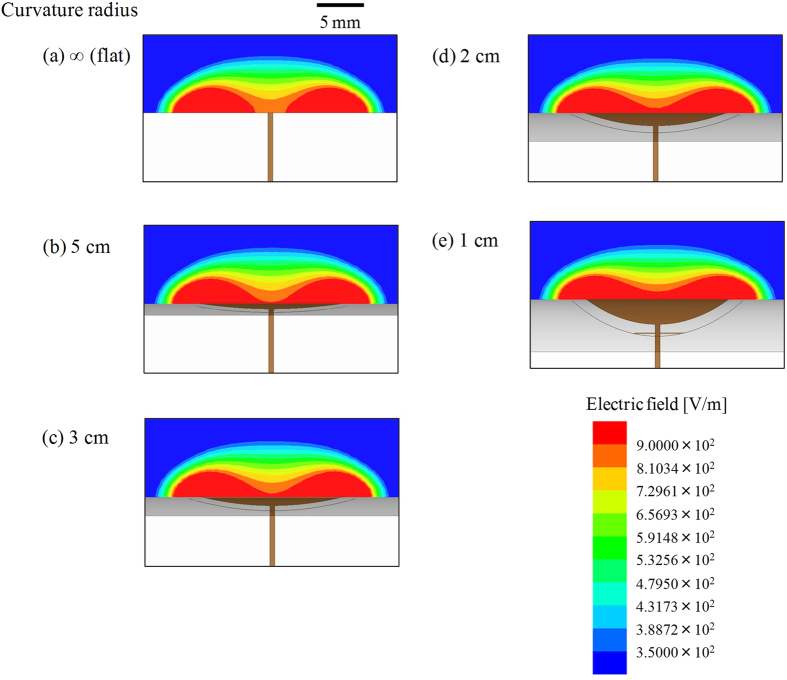
Simulation results of electric field distribution when the sensor is curved. (**a–e**) correspond to the curvature radii of ∞ (flat), 5, 3, 2, and 1 cm, respectively.

Figure 10a–e show the calculation results obtained for the sensor with *r*_t_ = 7.5 mm and *r*_b_ = 9 mm, curved with curvature radii *r* of ∞ (flat), 5, 3, 2, and 1 cm, respectively, which corresponded to the experimental results shown in Fig. 5. Note that in our simulation, the leader lines were halfway bent in the vertical direction. The region where *E* >9 × 102 V/m (red) obtained against the curved sensors seemed larger than with the sensor placed flat. This seemed consistent with the experimental results where the capacitance change in the curved sensors, except when *r* = 1 cm, was larger than that in the flat sensor in the range *d* ~ 1 mm (Fig. 5). Further, care should be taken to ensure that the distance between the top electrode along the *y*-axis shown in Fig. 1 and the approaching metal plate never reduces to zero because the sensor sheet was curved. The distance increased with decreasing curvature radius. This means that capacitance change became small when the curvature radius was low. Experimental results depicted in Fig. 5 show that capacitance change was small, along the order of *r* = 1, 2, 3, and 5 cm. Hence, it appears that there exists a tug-of-war of sorts in the sensor response in case of the curved sensor: an improvement in the intensity of the electric field, and degradation due to an increase in the distance between the electrode along the *y*-axis and the metal plate.

**Figure 11:**
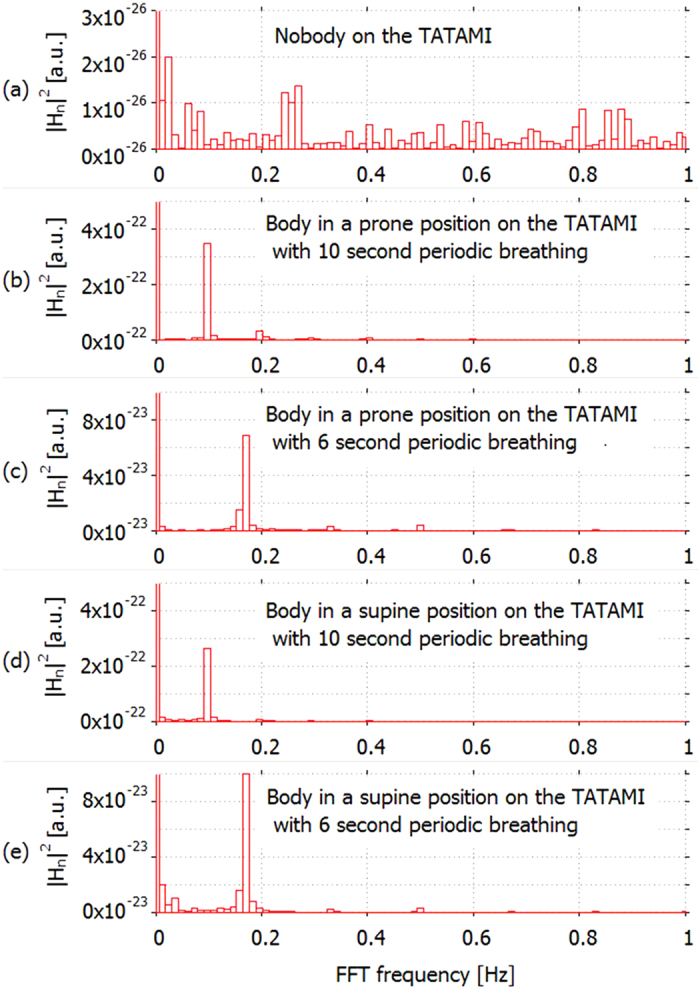
Calculation of frequency for visualization of the breathing period. Frequency obtained by using the fast Fourier transform (FFT). The graphs (**a**–**e**) correspond to the data shown in Fig. 7a–e, respectively: no one on the *tatami* (**a**), the examinee on the *tatami* in a prone position with 10-s periodic breathing (~4 s for suction, ~2 s for delivery, and 4 s for apnoea) (**b**), a prone position with 6-s periodic breathing (~3 s for suction and ~3 s for delivery) (**c**), a supine position with 10-s periodic breathing (~4 s for suction, ~2 s for delivery, and ~4 s for apnoea) (**d**) and a supine position with 6-s periodic breathing (~3 s for suction and ~3 s for delivery) (**e**).

In order to gain a better insight into the characterization of the data shown in Fig. 7, we used a Fourier transform to obtain frequency information. Figure 11a–e show the calculated frequency strength corresponding to Fig. 7a–e, respectively, by using the fast Fourier transform (FFT). The horizontal axis shows FFT frequency *f*_*n*_ displaying a frequency range in the region of 1 Hz, where *n* is the sample number of the FFT, detailed later in the ‘Methods’ section. The vertical axis shows the signal strength for each value of frequency. Note that the strength of the DC component (0 Hz) was greater than 2.4 × 10^−16^ for each case shown in Fig. 11a–e. No peaks can be seen when no one was on the *tatami* (Fig. 11a). Furthermore, the absolute value of the signal shown in Fig. 11a is quite small compared to other conditions under which the person lay on the *tatami* (Fig. 11b–e). On the contrary, apparent peaks were observed at 0.098 Hz (Fig. 11b,d) and 0.170 Hz (Fig. 11c,e). These frequencies agreed well with values of 0.1 Hz and 0.166 Hz, which corresponded to 10-s and 6-s breathing, respectively.

The results of our experiment indicate that our proposed sensor component has potential to detect human breathing, and the FFT technique helps understand this phenomenon intuitively. Moreover, we emphasize again that our sensor is not a contact-type sensor, but a non-contact-type sensor. Such a proximity sensor can be blended into surrounding environments. This suggests that the sensor can be used without being noticed by users, hence eliminating any discomfort for them. We think that systems based on our sensor can be widely used to inspect diseases, such as sleep apnoea syndrome, in the near future. We also stress that our proposed duplex screen/screen-offset printing was effective from the perspective of the fabrication of the arrayed sensor. It is believed that the development of such a two-dimensional sensor is very important because more advanced systems such as three-dimensional spatial mapping can be realized. The one-pixel sensor used in our principle experiment can be fabricated by single-side printing. Recent research has reported a one-pixel pyroelectric sensor fabricated through one-side ink-jet printing^30^. However, the passive matrix two-dimensional sensor, such as a touch screen panel, is difficult to form using a single-side printing method, and the ink jet requires lengthy fabrication takt time. Furthermore, in the fabrication of the arrayed sensor, electrode size should be taken into account. The results shown in Fig. 3 imply that larger values of *r*_b_ yield greater changes in capacitance, indicating that the larger sensor is superior to smaller sensors in terms of sensitivity. However, if we expand this sensor to two-dimensional sensor arrays, large values of *r*_b_ should result in reduced resolution. Moreover, appropriate electrode sizes vary according to whether the value of *d*_d_ needed or the desired sensitivity in the vicinity of the sensor (Δ*C*_0.1_) needed. We might have to take care in determining the proper electrode size depending on the application of the sensor. As the next step in this project, we will attempt to develop such a matrix-type sensor, and will elucidate the dependencies of sensor response and resolution on sensor electrode size. We will also try to clarify curvature dependence, and apply our sensor component to various situations.

One of the other concerns of the sensor is stability against human-related or environmental factors such as (i) humidity of air, (ii) human skin conditions and (iii) metallic wearable accessories. (i) Humidity: Humidity or other environmental conditions may affect the signals. Therefore, it may be effective to prepare another proximity sensor as a reference sensor, and set it at the place where humans cannot reach it; differential data between the detection sensor and the reference sensor should help decrease the disturbance. (ii) Human skin conditions: If rigorous measurement is necessary, we should perform the calibration using instruments such as a skin moisture analyser. Namely, the dependence of Δ*C* of the sensor on the skin condition is first obtained in advance, and the skin condition is measured before using the proximity sensor. During the measurement, the calibration is performed by these preliminary obtained data. Most of these analysers detect the skin moisture level using the electric field^38^. Although we can use a conventional instrument, our developed proximity sensor can also be used for skin moisture level detection. (iii) Metallic wearable accessories: It seems very difficult to eliminate the influence caused by metallic accessories. First, we should investigate the extent to which such an accessory affects the signals. However, as far as our developed proximity sensor is used as a bed sensor, it may not be a significant problem; most people remove such accessories when sleeping. Further, we will investigate these points as a future work.

## Methods

### Materials for screen and screen-offset printing

We used the screen printing machine Cube-1515 (Mino Group, Gifu, Japan). The ink used was flake-type Ag ink (trial product, Mino Group, Gifu, Japan). We used screen masks (Murakami, Chiba, Japan) with a mesh count of 500 inches^−1^, and its emulsion thickness was 10 μm. The silicone resin used was polydimethylsiloxane (PDMS, Shin-Etsu Chemical, Gunma, Japan) with a thickness of approximately 2 mm. A stainless steel plate was adhesively placed under the PDMS for reinforcement. The film sheet employed as a substrate of a proximity sensor was the PET film (Cosmoshine A4300, Toyobo, Shiga, Japan) with a thickness of 125 μm. Following printing, the ink patterns were sintered at 130 °C for five minutes.

### Fast Fourier transform (FFT)

We show the procedure used to calculate the spectrum strength in Fig. 11a–e. Raw capacitance data from the chemical impedance analyser, shown in Fig. 7a–e, were obtained in periods of ~10 ms, but not strictly 10-ms periods. Thus, we first linearly interpolated two adjacent data items, and generated a new data item at time intervals of 10 ms. A total of 8192 data items from the data head, namely, data in the first 81.92 s, were used to perform the FFT, and the complex amplitude of the discrete frequency from 0 Hz to 50 Hz was calculated at a frequency interval of 0.0122 Hz. Finally, the spectrum strength was calculated, the detailed procedure for which is explained as follows. We assumed that the time series data *h*_*k*_, at the time *t*_*k*_ = *k*Δ*t* were given against sampling point *k* ( = 0, 1, 2, …, *N*). In this case, the complex amplitude *H*_*n*_ against the discrete frequency *f*_*n*_ could be expressed as a discrete Fourier transform (DFT) as follows:


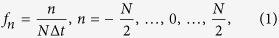



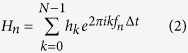


and the FFT was used to calculate *H*_*n*_^39^. In our case, *h*_*k*_ corresponded to the interpolated capacitance data, *N* = 8192, and Δ*t* = 10 ms, as mentioned above. The spectrum strength shown in Fig. 11a–e was calculated as the sum of the squares of each real and imaginary part of the complex amplitude *H*_*n*_, described by the following equation:


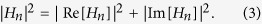


## Additional Information

**How to cite this article**: Nomura, K.-i. *et al.* A flexible proximity sensor formed by duplex screen/screen-offset printing and its application to non-contact detection of human breathing. *Sci. Rep.* **6**, 19947; doi: 10.1038/srep19947 (2016).

## Supplementary Material

Supplementary Information

Supplementary Movie

Supplementary Dataset S1

Supplementary Dataset S2

Supplementary Dataset S3
