# Supplementary material for: A flexible proximity sensor formed by duplex screen/screen-offset printing and its application to non-contact detection of human breathing
Source: Sci Rep. 2016 Jan 22;6:19947. doi: 10.1038/srep19947 (PMC4726207; doi:10.1038/srep19947)
Supplement: Supplementary Information [file srep19947-s1.pdf]

Supplementary Information

A flexible proximity sensor formed by duplex screen/screen-offset printing and its application to non-contact detection of human breathing

Ken-ichi Nomura, Ryosaku Kaji, Shiro Iwata, Shinobu Otao, Naoto Imawaka, Ryosuke Mitsui, Junya Sato, Seiya Takahashi, Shin-ichiro Nakajima, and Hirobumi Ushijima

Supplementary Movie

Demonstration of the proposed proximity sensor. In the demonstration, a metal rod, a dielectric rod, and a human hand approached the sensor. The responses of the sensor to the metal rod and the human hand were clearly observed due to their conductive properties, whereas the sensor was insensitive to the dielectric rod.

Supplementary Datasets

Raw data representatively selected from the circular plots (r_t = 0.5 mm) in Fig. 2(b). d = 0.1 (S1), 5 (S2), and 100 mm (S3).
